# Current insights into the implications of m6A RNA methylation and autophagy interaction in human diseases

**DOI:** 10.1186/s13578-021-00661-x

**Published:** 2021-07-27

**Authors:** Xuechai Chen, Jianan Wang, Muhammad Tahir, Fangfang Zhang, Yuanyuan Ran, Zongjian Liu, Juan Wang

**Affiliations:** 1 Center of Excellence for Environmental Safety and Biological Effects, Beijing International Science and Technology Cooperation Base for Antiviral Drugs, Faculty of Environment and Life, Beijing University of Technology, 100 Ping Le Yuan, Chaoyang District, Beijing, 100124 People’s Republic of China; 2 Department of Rehabilitation, Beijing Rehabilitation Hospital, Capital Medical University, Xixiazhuang, Badachu, Beijing, 100144 People’s Republic of China

**Keywords:** m6A, RNA methylation, Autophagy, Obesity, Azoospermatism, Ischemic heart disease, Cancer

## Abstract

Autophagy is a conserved degradation process crucial to maintaining the primary function of cellular and organismal metabolism. Impaired autophagy could develop numerous diseases, including cancer, cardiomyopathy, neurodegenerative disorders, and aging. N6-methyladenosine (m6A) is the most common RNA modification in eukaryotic cells, and the fate of m6A modified transcripts is controlled by m6A RNA binding proteins. m6A modification influences mRNA alternative splicing, stability, translation, and subcellular localization. Intriguingly, recent studies show that m6A RNA methylation could alter the expression of essential autophagy-related (*ATG*) genes and influence the autophagy function. Thus, both m6A modification and autophagy could play a crucial role in the onset and progression of various human diseases. In this review, we summarize the latest studies describing the impact of m6A modification in autophagy regulation and discuss the role of m6A modification-autophagy axis in different human diseases, including obesity, heart disease, azoospermatism or oligospermatism, intervertebral disc degeneration, and cancer. The comprehensive understanding of the m6A modification and autophagy interplay may help in interpreting their impact on human diseases and may aid in devising future therapeutic strategies.

## Background

Autophagy is an evolutionarily conserved mechanism that widely occurs in eukaryotic organisms. It attracted increasing attention due to its significant role in cell survival (during the state of energy or nutrient deficiency) and removing dysfunctional or unnecessary organelles and proteins [1]. In humans, aberrant autophagy regulation could develop various pathophysiological conditions, including cancer, aging, neurodegenerative disorders, and cardiomyopathy [2]. Autophagy occurs in three different forms: macro-autophagy, micro-autophagy, and chaperone-mediated autophagy (CMA) [3]. All the three forms follow the autophagy-lysosomal pathway (ALP), in which cytosolic material is transported to lysosomes for degradation. In macro-autophagy, autophagosome containing cytosolic components transports to the lysosome. Following its attachment to the lysosome, cytosolic components are degraded. In micro-autophagy, lysosomal membrane invaginates and cytoplasmic components are engulfed directly by the lysosome and degraded. In CMA, chaperone proteins (such as Hsc-70) make a complex with target proteins and enable their entry into the lysosomes through the lysosomal-associated membrane protein 2A (LAMP-2A) receptor. Following entry, target proteins are finally degraded [4].

**Fig. 1:**
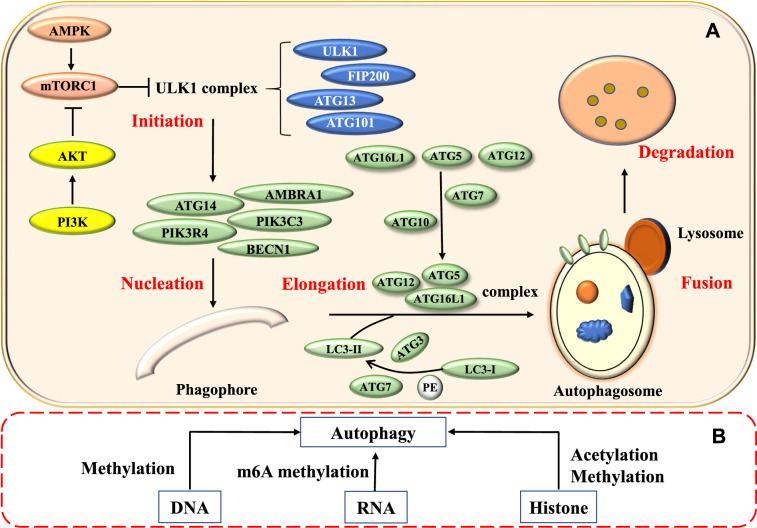
The process of macro-autophagy and its epigenetic regulation. **A** Autophagy is initiated with the formation of ULK1 complex (ULK1-ATG13-FIP200). BECN1, ATG14 and PI3K complex facilitate the formation of phagophore. ATG5-ATG12-ATG16 complex and PE-conjugated-LC3II promote phagophore elongation and autophagosome formation. Autophagosome fusion with the lysosome results in the degradation of target molecules. **B** Epigenomic modifications in DNA, RNA, and histones regulate autophagy by modifying the expression of autophagy-related genes or affecting the autophagy-associated signaling pathways

Macro-autophagy (henceforth referred to as autophagy) is the most prevalent form of autophagy (Fig. 1A). It starts with the formation of autophagosome (double-membrane-bound vesicle), which harbors the target cellular components. Autophagosomes deliver the unwanted cellular components to the lysosome for degradation by lysosomal hydrolases. Numerous autophagy-related (*ATG*) genes regulate this whole process. Several factors contribute to initiating the cytoprotective autophagy process. Nutrient deficiency, oxygen depletion, and harmful proteins produce stress condition, which inactivates the mTOR (mammalian target of rapamycin) complex. Therefore, Unc-51 like kinase 1/2 (ULK1/2) is activated. The activated ULK1/2 kinase promotes the binding of the focal adhesion kinase family interacting protein of 200 kDa (FIP200) to the ATG13 protein. ATG13-FIP200 complex further phosphorylates ULK proteins. Subsequently, ATG13-FIP200 complex and phosphorylated ULK proteins recruit more ATG proteins and facilitate the formation of double-membrane autophagosome [5–8]. Afterwards, the autophagosome moves to the lysosome with the help of microtubule proteins. LC3-II is one of the LC3 (microtubule-associated protein 1A/1B-light chain 3) proteins. It facilitates the fusion of the autophagosome to the lysosome to form autolysosomes, and this dynamic process is called autophagic flux [9].

Many studies indicate that epigenetic modifications such as DNA methylation, histone modifications and RNA modifications play a vital role in autophagy regulation [10, 11] (Fig. 1B). Such modifications can directly influence the expression of *ATG* genes or interfere with signaling mechanisms that regulate autophagy.

N6-methyladenosine (m6A), characterized by adenosine methylation at nitrogen 6 position, is one of the most profound post-transcriptional modifications. It commonly occurs in mRNAs and long non-coding RNAs (lncRNAs) in higher eukaryotes and is considered the predominant internal modification in RNA. m6A functionally regulates the eukaryotic transcriptome by influencing mRNA splicing, export, subcellular localization, translation, stability, and decay. Thus, aberrant m6A methylation could modulate biological processes and develop human diseases [12].

Recently, one report showed that the post-transcriptional regulation of Atg1/ULK1 could be altered by m6A RNA modification, resulting in autophagy inhibition [13]. Thereafter, many studies demonstrated the effects of m6A modification in the autophagy mechanism [14–16]. In some cases, the m6A modification imparts direct inhibitory effects on autophagy [17]. It could also affect the formation of autophagosomes to dysregulate autophagy [18]. Sometimes it could promote autophagy initiation [19]. The current data shows that the m6A modification plays a crucial role in regulating autophagy. Moreover, the effects of the m6A modification on autophagy are disease context-dependent. Since both m6A modification and autophagy play critical roles in regulating health conditions, this review summarizes the inferences of the latest studies, which explored the effects of m6A modification and autophagy interactivity on human diseases, including obesity, heart disease, fertility disorders, intervertebral disc degeneration, and cancer. A comprehensive understanding of the m6A and autophagy relationship in human diseases may benefit in devising therapeutic strategies in the future.

## Reversible/dynamic m6A RNA methylation in autophagy modulation

**Fig. 2:**
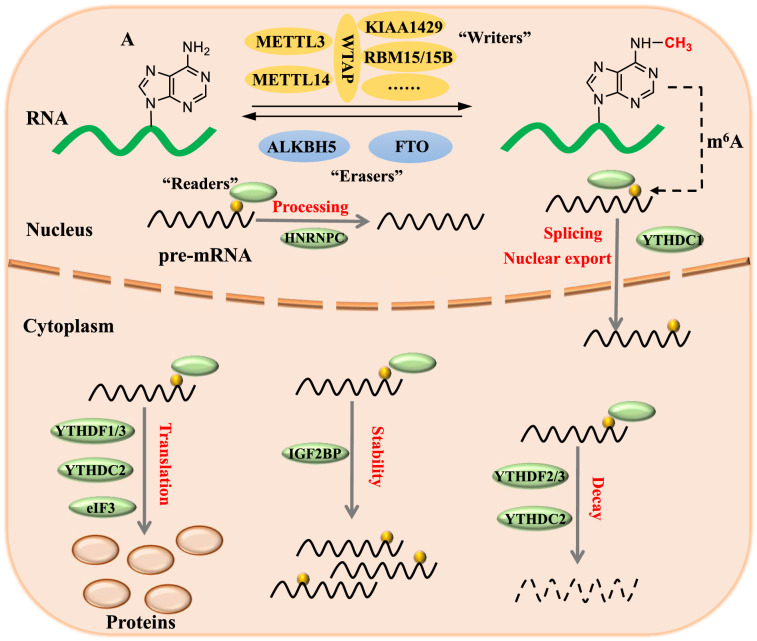
The dynamic and reversible m6A RNA modification and its role in RNA metabolism. m6A RNA methylation is exerted by methyltransferases (METTL3, METTL14, WTAP, etc.), removed by demethylases (FTO and ALKBH5). mRNA processing, splicing, stability, translation, and subcellular localization can be influenced by m6A modification through the actions of m6A binding proteins

It is observed that one-fourth of the cellular transcriptome contains multiple m6A modification residues [20, 21]. Furthermore, it is identified that the RRACH motif (R=A or G, H=A, C, or U) in RNA is the primary site for m6A modification [22–24]. The m6A modification is dynamic and reversible and is regulated by various protein complexes [25, 26]. m6A modification is exerted by methyltransferases (Writers) [27, 28], eliminated by demethylases (Erasers) [29, 30], and recognized by m6A binding proteins (Readers) (Fig. 2).

m6A modification and its regulatory enzyme complexes play an important role in the mRNA life cycle. Methyltransferases commonly exerting m6A modification include methyltransferase-like 3 (METTL3), methyltransferase like-14 (METTL14), vir-Like m6A methyltransferase associated (VIRMA, KIAA1429), RNA binding motif protein 15 (RBM15), and Wilms’ tumor 1-associating protein (WTAP) [31, 32]. m6A methylation process is initiated when METTL14 binds to METTL3 to constitute a stable heterodimer core complex [33]. METTL3/ METTL14 methyltransferase complex and WTAP carry out the deposition of m6A on nuclear RNA in mammalian cells [28]. Studies reported that RBM15 and KIAA1429 facilitate the METTL3/ METTL14/ WTAP complex to induce m6A modification. Decreased KIAA1429 and RBM15 protein levels reduced the deposition of m6A on mRNA [34, 35]. This finding is suggestive of their critical role in the methylation process. Besides, other methyltransferases were also discovered recently, such as METTL16 works independently to induce m6A deposition on nuclear RNA [32], and METTL5 induces m6A on ribosomal RNA [36].

m6A modification is reversible due to the two demethylases, including fat mass and obesity-associated protein (FTO) and alpha-ketoglutarate-dependent dioxygenase alkB homolog 5 (ALKBH5) proteins. These are mainly present in the nuclear compartments [29, 30]. As identified for the first RNA demethylase, FTO removes methyl group of m6A and is essential for mRNA processing. ALKBH5 could also eliminate m6A modification from mRNA. It also has a profound role in nuclear RNA export and metabolism [30, 37].

Proteins are defined as “Readers”, which selectively bind to m6A modified sites on mRNA. Readers regulate m6A modification by altering the recognition of modified mRNA [38]. YTH-domain N6-methyladenosine RNA-binding proteins (YTHDF1, YTHDF2, YTHDF3, YTHDC1, and YTHDC2) are the prominent reader proteins. YTHDF1 enhances m6A mRNA translation by promoting ribosome assembly and making interaction with the initiation factor. YTHDF2 attenuates the stability of m6A RNA and promotes its degradation by directing it to processing bodies (P bodies) in the cytoplasm. YTHDF3 facilitates YTHDF1 and YTHDF2 to execute their functions [39–44]. YTHDC1 regulates pre-mRNA splicing and RNA exportation, while YTHDC2 directly interacts with ribosome subunits. Hence, it interferes with mRNA translation. Besides the YTH-domain protein family, other proteins such as insulin-like growth factor 2 mRNA-binding proteins (IGF2BPs) also bind to m6A modified sites and enhance mRNA stability [45].

Therefore, “Writers”, “Erasers”, and “Readers” dynamically regulate m6A modification. Being the most abundant mRNA modification, m6A could modulate various biological processes, including autophagy.

Autophagy is a lysosome-assisted degrading mechanism that helps the cells to cope with stress conditions [46, 47]. Recently, several studies determined the potent role of m6A modification in autophagosome formation and autophagy regulation [48]. m6A modification could influence the transcriptional regulation of ATG proteins and affect the autophagy mechanism.

The mechanistic target of rapamycin complex 1 (mTORC1) could inhibit autophagy through phosphorylation of Atg13. A report showed that mTORC1 could activate the chaperonin containing tailless complex polypeptide 1 (CCT) to stabilize methyltransferase complex (METTL3/ METTL14). As a result, m6A levels increased on the mRNAs of *ATG* genes, and the transcripts of these genes became highly susceptible to degradation. Hence autophagy is suppressed [49]. Moreover, another study revealed that decreased levels of METTL14 contribute to promoting autophagy in Leydig cells (LCs) [48]. This study showed that reduction in METTL14 levels provided stability to the mRNA of calcium/calmodulin-dependent protein kinase kinase 2 (CAMKK2). Subsequently, CAMKK2 activated the adenosine 5-monophosphate-activated protein kinase (AMPK) and ULK1 complex (positive regulators of autophagy), which initiated the autophagy. FTO demethylase was also observed to promote autophagy by splitting ULK1 from YTHDF2, thus increased ULK1 expression [13]. Observations in ovarian cancer cells showed that ALKBH5 inhibits autophagy. Reduction in ALKBH5 expression resulted in degradation of BCL-2 mRNA. Consequently, the BCL-2-Beclin1 complex (negative regulators of autophagy) was disrupted, and autophagy was activated [50].

Upon FTO depletion, m6A modification of *ATG7* and *ATG5* mRNAs happens directly, which provides a basis for binding YTHDF2 protein to the *ATG* transcripts. This binding ultimately dysregulates the autophagosome assembly [18]. It is also reported that upregulation of METTL3 induced methylation and triggered the binding of YTHDF1 to forkhead box class O3 (FOXO3) transcripts and provided stability to the FOXO3 mRNA. Hereafter, FOXO3 halted the expression of *ATG* genes to inhibit autophagy [51]. Furthermore, METTL3 mediated m6A modification of transcription factor EB (TFEB) mRNA resulted in its binding to m6A reader protein heterogeneous nuclear ribonucleoprotein D (HNRNPD). TFEB is considered a major transcriptional regulator of lysosome biosynthesis and autophagy. The binding of HNRNPD to TFEB reduced TFEB levels, which resulted in decreased lysosome biosynthesis and impaired autophagy [17, 47, 52].

As one of the most prevalent RNA modifications, m6A plays an important role in the regulation of the stability and translation of mRNAs, and is involved in various bioprocesses. m6A RNA modification could regulate autophagy by modifying the expression of *ATG* genes or affecting the autophagy-associated signaling pathways, hence regulates various physiological and pathological processes. Taken together, the influence of the m6A modification on autophagy is complex and dynamic, and its regulatory mechanism needs to be further determined.

## m6A-autophagy regulation in metabolic related diseases

### Role of m6A modification and autophagy interactivity in adipogenesis and obesity

For the last few decades, obesity and its related disorders are emerging worldwide [53, 54]. Obesity is characterized as an irregular or unhealthy accumulation of adipose tissue due to an increase in adipocyte volume (hypertrophy) or amount of fatty tissue (hyperplasia). Several studies confirmed that numerous biological processes control adipogenesis, including transcriptional mechanisms, and epigenetic alterations [55].

As the most prevalent eukaryotic mRNA modification, m6A could influence adipogenesis [56–59]. FTO plays a critical role in regulating fat mass and body weight, and m6A levels are inversely linked to adipocyte differentiation [60, 61]. Likewise, autophagy is also known to regulate fat mass accumulation and lipogenesis [62]. Empirical evidence suggests that obesity could occur due to compromised autophagy [63, 64]. Excessive consumption of nutrients promotes obesity and triggers mTORC1 activity. Consequently, the synthesis of many ATG proteins is inhibited, which leads to the suppression of autophagy [65]. It is also reported that adipose tissue mediated lysosomal dysfunction could cause autophagosome retention and lower autophagic clearance [66].

Numerous studies have examined the interaction between FTO and autophagy. FTO can act as an amino acid sensor, and it can significantly improve the functioning of mTORC1 and regulate autophagy [67–69]. Aas et al. showed that autophagy remained unaffected in response to upregulation of FTO in nutrients depleted U2OS cells [70]. On the contrary, research conducted in MEF cells exhibited that arsenic-mediated upregulation of FTO inhibited autophagy, and autophagy inhibition could increase the stability of FTO [71]. Previously, it also reported that FTO knockdown in Hela cells could inhibit autophagy by downregulating the expression of ULK1 [13]. Taken together, the diversified role of autophagy attributes to producing various outcomes in different tissues or cells. The variations in FTO-mediated autophagy could presumably be asserted by the specific form or state of cells used in the experiments.

**Fig. 3:**
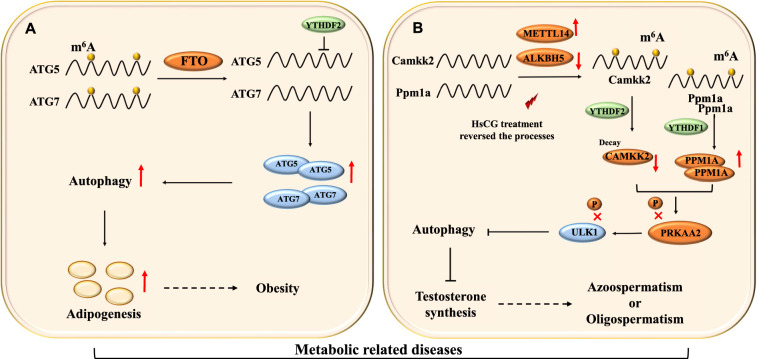
Effects of m6A-autophagy interaction in metabolic related diseases. **A** FTO-mediated demethylation provides stability to ATG5 and ATG7 transcripts. Subsequently, autophagy is induced, which promotes adipogenesis and obesity. **B** m6A mediated reduction in AMPK (AMP-activated protein kinase) activity resulted in autophagy inhibition. Consequently, testosterone synthesis decreased, which mediated azoospermtism or oligospermatism

One recent study in adipocytes reported that ATG5 and ATG7 proteins play a vital regulatory role in FTO-mediated autophagy (Fig. 3A) [18]. ATG7 conducts ATG12-ATG5 covalent binding via a ubiquitin-like conjugation mechanism. The resulting ATG12-ATG5 homodimer attaches to ATG16L and facilitates autophagosome elongation [72, 73]. It is observed that FTO depletion decreased the ATG12-ATG5 covalent binding, reduced ternary complex development, and attenuated the autophagy activation. This FTO-mediated attenuation of autophagy and *ATG* genes expression is observed to be associated with m6A modification [18].

On the contrary, a recent study reported that FTO has no impact on ULK1 protein levels in preadipocytes [18]. This result contradicts the findings reported in a previous study in HEK293T cells, which showed FTO could alter the ULK1 protein levels [13]. The study in preadipocytes exhibited that FTO knockdown or overexpression failed to alter the ULK1 protein levels. Moreover, this study reported that YTHDF2 overexpression reduced the autophagy-related protein levels by targeting the m6A-modified mRNAs of *ATG* genes. Forced expression of YTHDF2 failed to modulate ULK1 mRNA or protein levels and autophagy in preadipocytes. The reason for this outcome might be the inability of YTHDF2 to recognize ULK1 mRNA in adipocytes specifically. These outcomes exhibited that FTO-mediated alterations in m6A modification and activity of YTHDF2 influence the expression of ATG proteins and autophagy process in cell type-specific manner [18].

The findings provide an understanding of m6A, and autophagy regulated mechanisms of adipogenesis. These could benefit in identifying targets to combat obesity and its associated health issues.

### Role of m6A modification and autophagy interplay in male fertility disorders

Spermatogenesis is a complex process responsible for the morphological and biochemical changes in spermatogenic stem cells (SSCs), which develop into elongated mature spermatozoa. Spermatogenesis is regulated through numerous transcriptional, posttranscriptional, and translational processes [74]. Many hormones perform their vital role in spermatogenesis, especially testosterone plays a crucial role in this process [75, 76]. The Leydig cells (LCs) found in the testis interstitium are the primary site for the synthesis of testosterone in males. In the absence of testosterone, spermatogenesis halts at the meiosis stage. Thus, deficiency of testosterone could cause degeneration of germ cells at post meiosis. Furthermore, mature sperms could stay within the Sertoli cells, leading to azoospermia, oligospermia, or infertility.

Literature review revealed that autophagy is a critical regulatory process in testosterone synthesis and spermatogenesis [77, 78]. Huang et al. demonstrated that autophagy core protein ATG5 is vital for male fertility due to its role in spermatogenesis [79]. Autophagy begins in the forerunner LCs, is steadily enhanced with LCs differentiation, and culminated in mature LCs. These findings imply that autophagy activity constantly changes during LCs differentiation.

Emerging evidence shows that m6A alteration could affect the gene expression in male germline cells [80, 81]. Recently, a study showed that m6A modification levels steadily decreased in LCs during their transformation from stem LCs into mature LCs. This finding indicates a potential role of m6A in LCs differentiation. Furthermore, this study also showed that m6A could negatively impact autophagy in LCs [48]. m6A modification was observed to attenuate ULK1 and TFEB (transcriptional regulators of autophagy) mRNA levels, which resulted in autophagy inhibition in LCs [13, 17].

In a recent study, LCs were treated with human chorionic gonadotropin (HsCG) to investigate autophagy dependency on AMPK-ULK1. The upstream kinases such as STK11/LKB1 and CAMKK2 could increase, and PPM1A phosphatase could reduce the phosphorylation of AMPK. HsCG treatment in LCs enhanced the expression of CAMKK2 kinase and reduced the level of PPM1A phosphatase, which facilitated the activation of PRKAA2 mediated autophagy (Fig. 3B) [48]. Further experiments demonstrated that m6A could interfere with PRKAA2 activity by enhancing PPM1A translation and CAMKK2 mRNA degradation in an m6A-dependent manner [48]. HsCG treatment caused a reduction in m6A modification on CAMKK2 and PPM1A transcripts, which resulted in decreased PPM1A levels and increased CAMKK2 levels. Moreover, upregulation of PPM1A and depletion of CAMKK2 resulted in attenuation of HsCG-triggered autophagy in LCs. This finding suggests that both PPM1A and CAMKK2 are essential for autophagy induction, and synchronized regulation of these proteins could provide a possibility to control testosterone synthesis.

m6A modification could alter testosterone synthesis and develop oligospermia or azoospermia. These findings emphasize the essential role of m6A RNA modification in the regulation of autophagy and testosterone synthesis. These findings suggest that new therapeutic strategies can be developed by targeting m6A RNA modification in patients with testosterone deficiency, azoospermia, and oligospermia.

## m6A-autophagy regulation in apoptosis-induced diseases

### Role of m6A modification and autophagy interactivity in cardiomyocytes apoptosis and ischemic heart disease

Cardiovascular diseases (CVDs) are among the common causes of human illness and death in the world. Several studies reported irregular m6A methylation could promote CVDs incidence, including ischemic heart disease, cardiac arrest, cardiac hypertrophy [82, 83]. It is observed that autophagosome formation increases during ischemia and reperfusion, and the AMPK might be responsible for this increment [84]. Trehalose (a disaccharide) upregulates TFEB and stimulates autophagy, and prevents cardiomyocyte apoptosis [85]. Cardiomyocytes can keep their mitochondria healthy by mitophagy, which protects the heart from ischemic injury [86]. This data suggest that autophagy could prevent ischemic heart disease, but the underlying molecular mechanisms still need to be elucidated.

**Fig. 4:**
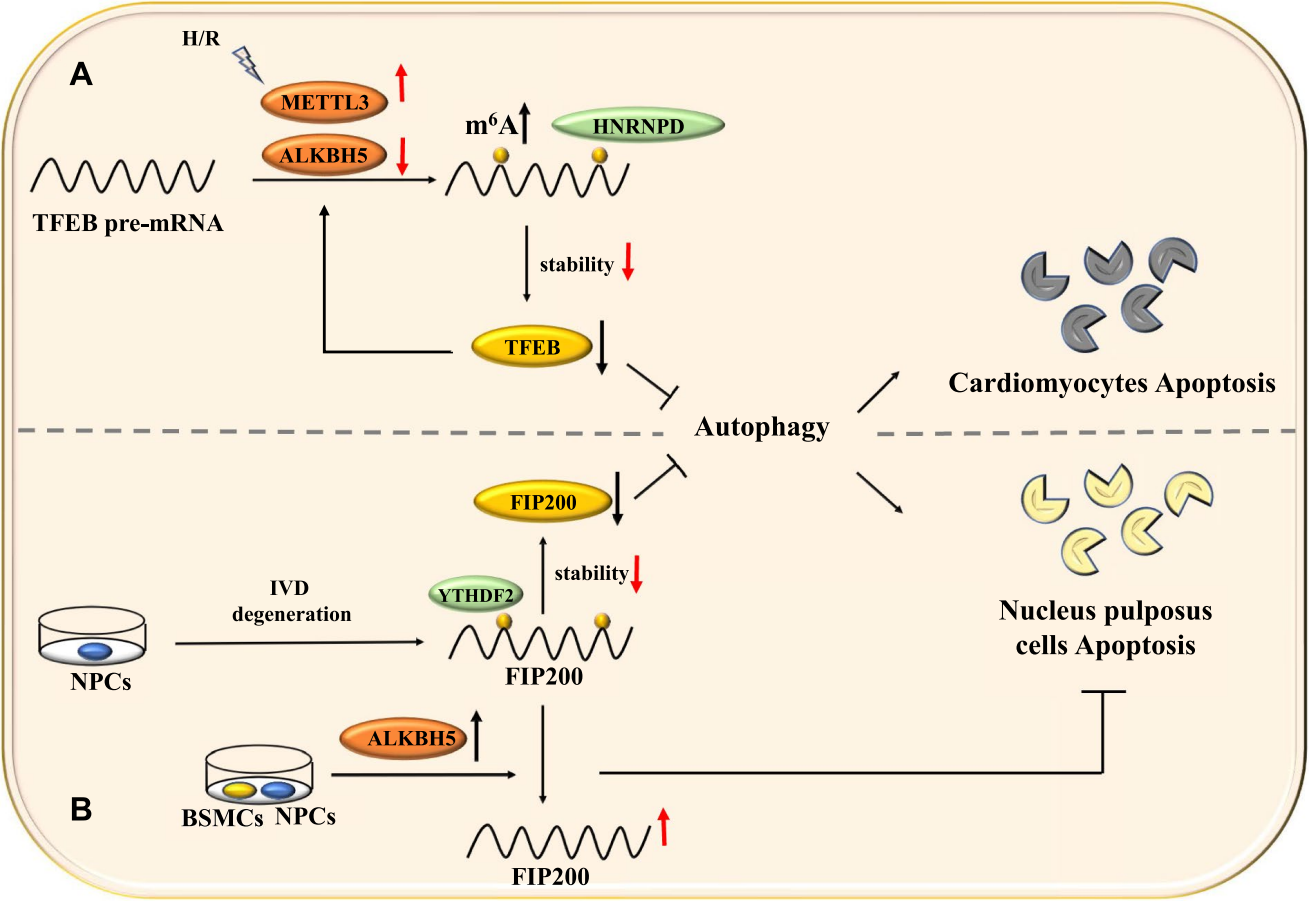
Effects of m6A-autophagy interaction in apoptosis-induced diseases**. A** Hypoxia/ Reoxygenation (H/R) mediated increased m6A modification could provide stability to TFEB transcripts, promoting autophagy and inhibiting apoptosis of cardiomyocytes. On the contrary, HNRNPD could reduce TFEB mRNA stability which could result in autophagy inhibition and apoptosis induction in cardiomyocytes. **B** YTHDF2 mediated degradation of FIP200 mRNA causes autophagy inhibition and apoptosis induction in NPCs. BSMCs-NPCs co-culturing could induce autophagy which prevents apoptosis of NPCs

m6A may be a new starting point to analyze the regulatory processes of autophagy in heart disease (Fig. 4A). Previous studies showed that the FTO-dependent m6A pathway plays a critical role in cardiac remodeling and restoration [87]. Song et al*.* investigated the function of m6A regulated autophagy in hypoxia/reoxygenation (H/R) cardiac muscle cells [17]. m6A modification significantly increased in H/R-treated cardiomyocytes and ischemia/reperfusion (I/R)-treated mice heart, and it occurred due to the elevated expression of METTL3 and decreased expression of ALKBH5. METTL3 is highly expressed in cardiomyocytes during H/R therapy, which could interfere with autophagic flux in the cardiomyocytes. METTL3 mediated increased m6A modification caused HNRNPD binding to TFEB pre-mRNA and reduced its stability. As a result, the TFEB level decreased. TFEB is a key regulator of autophagy [88]. Its deficiency could reduce autophagy activity in cardiomyocytes, resulting in increased apoptosis in these cells [89]. Song et al*.* reported that METTL3 depletion in H/R-treated cardiomyocytes might improve cell viability. This finding suggests that the targeted inhibition of METTL3 may provide new avenues to formulate therapeutic strategies for cardiovascular diseases.

### Role of m6A modification and autophagy interplay in apoptosis of nucleus pulposus cells and intervertebral disc degeneration

Degenerative changes in nucleus pulposus cells (NPCs) could cause degeneration of the intervertebral disc (IVD). It is thought to be the most common cause of back pain. Studies showed that autophagy could reduce NPCs' degenerative changes, thus minimizing the risk of IVD degeneration [90].

Li et al*.* recently reported that bone marrow-derived mesenchymal stem cells (BMSCs) could promote autophagy and reduce apoptosis in NPCs by modulating m6A modification in a co-culture model [91]. During IVD degeneration, m6A modification of FIP200 mRNA occurs. YTHDF2 binds to m6A modified FIP200 transcripts and degrades them. BMSCs and NPCs co-culturing resulted in enhanced AKLBH5 expression, which demethylated the FIP200 mRNAs and prevented their degradation. Furthermore, the reduction in m6A modification of FIP200 mRNA led to a decrease in their YTHDF2-mediated degradation. Consequently, autophagy activity was enhanced, which reduced the risk of apoptosis in NPCs in the co-culture model. The findings offer a novel theoretical basis for reversing IVD degeneration (Fig. 4B).

## m6A-autophagy regulation in cancer

### Role of m6A modification and autophagy interactivity in cancer progression

Increasing evidence shows that m6A modification is associated with multiple human cancers, including breast cancer, lung cancer, and glioblastoma [92]. Autophagy is an intracellular clearance mechanism that is regulated by numerous proteins. It is observed to promote metastasis of malignant tumor cells. mTOR is a vital regulator of autophagy. mTOR is also a downstream target of the phosphatidylinositol 3-kinase (PI3K) and kinase AKT pathways. In endometrial cancer, m6A modification regulates AKT activity, which indicates that m6A can potentially influence mTOR regulation through the AKT signaling pathway [93]. However, the exact mechanism of coordination between m6A modification and autophagy and its effects on cancer progression need to be further explored.

**Fig. 5:**
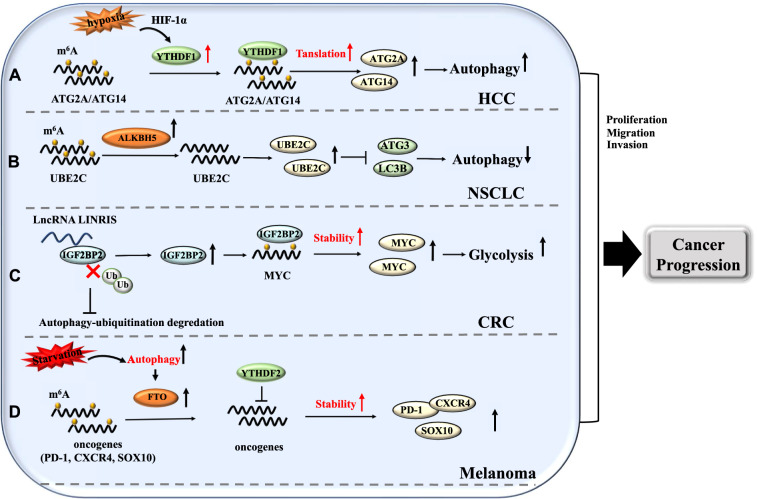
Effects of m6A-autophagy interaction in cancer progression. **A** HIF-1α induced YTHDF1 expression promotes ATG2A and ATG14 translation. Subsequently, autophagy is activated, which could promote HCC progression. **B** m6A provides stability to UBE2C transcripts, which could inhibit autophagy. Consequently, the proliferation, clonal expansion, and invasive growth of NSCLC are promoted. **C** IGF2BP2 mediated increased stability of MYC mRNA promotes glycolysis and cancer cell proliferation in CRC. **D** Autophagy-mediated upregulation of FTO could increase the stability of PD-1, CXCR4, SOX10 transcripts and which can promote the progression of melanoma

It is well known that hypoxia could promote the development and progression of cancers. Hypoxia can induce autophagy, which can help the cancer cells to cope with hypoxic conditions. Recently, a study reported that m6A reader YTHDF1 could promote hypoxia-induced autophagy, which in turn facilitated the development of human hepatocellular carcinoma (HCC) [94]. In hypoxia stress, HIF-1α can induce YTHDF1 expression, which promoted the translation of ATG2A and ATG14 in an m6A-dependent manner. The resulting hypoxia-induced autophagy then promoted the progression of HCC (Fig. 5A).

Guo et al*.* discovered that the ubiquitin-binding enzyme UBE2C is highly expressed in patients with non-small cell lung cancer (NSCLC), and UBE2C activation is one of the main factors that drives lung cancer incidence and metastasis [95]. It also reported that the expression of ALKBH5 is high in lung cancer cells. ALKBH5 knockdown reduced the levels of UBE2C, ATG3, and LC3 expression. NSCLC proliferation, clonal development, and invasion depend on the UBE2C-autophagy repression axis (Fig. 5B). In colorectal cancer (CRC), m6A reader protein IGF2BP2 could stabilize MYC mRNA, thus promote glycolysis [96]. As a result, the cellular energy increases, which promotes cellular proliferation. A lncRNA called LINRIS is highly expressed in CRC, which prevents IGF2BP2 destruction via the autophagy ubiquitination pathway [96] (Fig. 5C). The regulatory role of lncRNA in gene transcription and RNA stability has also been reported previously [97]. Patients suffering from melanoma are highly susceptible to developing resistance to conventional anti-cancer therapies. Yang et al. reported that FTO is essential for the progression of melanoma and the development of anti-PD-1 resistance [98]. In melanoma, starvation triggers autophagy and the NF-kB pathway, which in turn activates FTO. Mechanistically, FTO depletion elevates m6A-modification of PD-1, CXCR4, and SOX10 transcripts. Consequently, these transcripts are degraded by YTHDF2. These findings suggest that novel therapeutic strategies for melanoma can be devised by employing anti-PD-1 agents and FTO pathway inhibitory agents (Fig. 5D).

Given the results of several studies, it is conceived that m6A modifications and aberrant autophagy regulation could promote the incidence and progression of many types of cancer. Therefore, there is an utmost need to understand the molecular mechanisms which promote m6A-autophagy interaction mediated cancer development.

### Role of m6A modification and autophagy interplay in cancer drug resistance

**Fig. 6:**
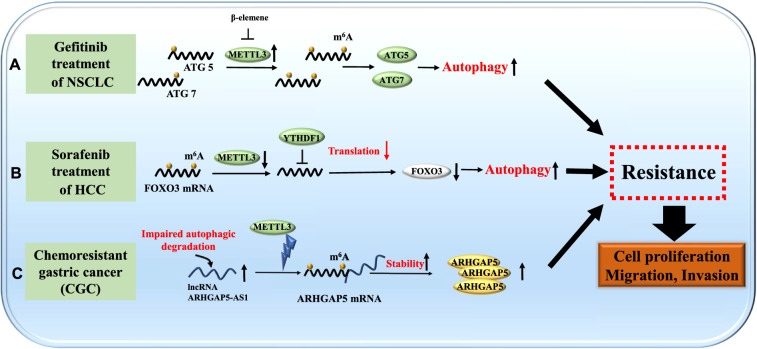
Effects of m6A-autophagy interaction in cancer drug resistance. **A** NSCLC could develop gefitinib resistance through m6A mediated activation of autophagy. **B** HCC could develop sorafenib resistance through m6A mediated decreased expression of FOXO3, which contributes to activation of autophagy. **C** Defective autophagy and m6A mediated increased stability of ARHGAP5 transcripts could promote chemoresistance in gastric cancer cells

Many recent studies exhibited that m6A modification mediated dysregulation of autophagy is well connected to the development of cancer drug resistance (Fig. 6). Gefitinib resistance is the main hurdle in achieving better therapeutic effects in NSCLC. Liu et al. discovered that β-elemene (an anti-cancer drug) could reverse gefitinib resistance through modulating METTL3-mediated autophagy [19]. This study showed that METTL3 could increase the expression of ATG5 and ATG7. Simultaneously, β-elemene attenuated m6A methylation of ATG transcripts by inhibiting METTL3 expression. Subsequently, it resulted in inhibition of autophagic flux and reversing gefitinib resistance in NSCLC (Fig. 6A).

HCC patients frequently receive sorafenib treatment. In the advanced stage of HCC, patients are highly susceptible to developing sorafenib resistance. Lin et al. demonstrated the essential role of METTL3-mediated m6A modification in the hypoxic tumor microenvironment and revealed that FOXO3 is primarily targeted by m6A modification in sorafenib-resistant tumors [51] (Fig. 6B). FOXO3 could reduce the expression of ATG proteins, including ATG5, ATG7, ATG16L1, and MAP1LC3B in HCC. These findings suggest that FOXO3 is vital for achieving m6A-dependent chemo-sensitivity in HCC due to its inhibitory effects on autophagy.

A recent study discovered the increased levels of ARHGAP5-AS1 (a lncRNA) in chemo-resistant gastric cancer (CGC) [99]. Its high expression resulted due to the impaired autophagy. ARHGAP5-AS1 could enhance the expression of ARHGAP5 (chemoresistance promoting gene) by stabilizing ARHGAP5 transcripts in CGC. ARHGAP5-AS1 mainly stabilizes ARHGAP5 mRNA by promoting METTL3 mediated m6A modification (Fig. 6C). These findings reveal that m6A modification and dysregulated autophagy contribute to attaining chemoresistance in CGC.

Taken together, the understanding of the role of m6A-autophagy interaction in cancer chemo-resistance might help in solving many unanswered questions and may provide opportunities to develop novel therapeutic strategies to overcome chemo-resistance in cancer.

## Conclusions and prospects

For the first time in 2018, Jin et al*.* reported a connection between m6A modification and autophagy [13]. Since that time, several studies have been conducted to understand the role of this relationship in various health conditions. Jin et al*.* showed that FTO knockdown downregulated the ULK1 abundance. Subsequently, it inhibited autophagy. This finding indicates that FTO is a positive regulator of autophagy. Another m6A demethylase, ALKBH5, also positively impacted autophagy [17, 48, 91]. Increased expression of ALKBH5 in NPCs in a co-culture model decreased m6A methylation on the FIP200 transcript and stabilized it, which ultimately enhanced the autophagy and inhibited the apoptosis [91]. These research outcomes revealed that both m6A demethylases (FTO and ALKBH5) could positively regulate autophagy and showed that m6A modification is inversely associated with the autophagy process.

Current research data demonstrated that m6A modification could influence autophagy initiation and elongation through regulating the expression of ULK1, FIP200, and ATG5, ATG7, respectively. Moreover, m6A modification was also observed to regulate the AMPK/AKT pathway, which has an essential role in autophagy regulation. m6A modification promotes PPM1A (AMPK negative regulator) expression and impedes the expression of CAMKK2 (AMPK positive regulator). Such alterations contribute to autophagy inhibition [48]. Moreover, reduced m6A modification levels could also activate AKT signaling pathways [100, 101]. AKT pathway is well known for promoting the incidence and progression of various diseases. Therefore, further investigations to explore the dynamic role of m6A modification in regulating the AKT pathway and expression of autophagy-related genes could provide new avenues for future studies.

Since both m6A epigenetic modification and autophagy play crucial roles in cellular and organismal metabolic activities, many studies conducted to explore the m6A-autophagy interaction in various human diseases (Table 1). m6A-autophagy interactivity could influence adipogenesis and testosterone synthesis, and induce obesity and male fertility disorders, respectively. Moreover, the m6A-autophagy interaction could induce apoptosis in cardiomyocytes and nucleus pulposus cells, which can cause ischemic heart disease and IVD degeneration, respectively. Given the vital role of autophagy in the onset of CVDs, further exploration of autophagy-related signaling pathways is needed. In addition, investigation of the regulatory role of m6A-autophagy interplay in cancer onset (such as liver cancer, gastric cancer, lung cancer) and cancer drug resistance is currently a popular area of research. Novel findings in this research area could help in devising treatment strategies to overcome cancer-related problems.

**Table 1:** m6A and autophagy associated factors involved in m6A-autophagy interaction and their potential mechanisms in human diseases

Human Diseases	m6A-associated factors	Autophagy-associated factors	Up/Down regulation of m6A methylation	Association between m6A modification and autophagy	Potential Mechanisms	References
Adipogenesis and Obesity	FTO	ATG5/ATG7	Up	Negative	YTHDF2-dependent ATG5/ATG7 mRNA degradation	[18]
Azoospermatism and oligo-spermatism	METTL14 /ALKBH5	AMPK regulator (PPM1A/ CAMKK2)	Up	Negative	m6A modification reduced AMPK activity	[48]
Ischemic heart disease	METTL3/ ALKBH5	TFEB	Up	Negative	HNRNPD-dependent TFEB decreased expression	[17]
IVD degeneration	ALKBH5	FIP200	Up	Negative	YTHDF2-mediated FIP200 mRNA degradation	[91]
HCC	YTHDF1	ATG2A/ATG14	/	/	HIF-1α-induced YTHDF1 expression promotes ATG2A/ATG14 translation	[94]
NSCLC	ALKBH5	UBE2C/ATG3/LC3	Down	Negative	ALKBH5 activated increases UBE2C-autophagy axis	[95]
CRC	IGF2BP2	Ubiquitin-autophagy pathway	/	/	IGF2BP2 increases MYC mRNA stability	[96]
Melanoma	FTO	Metabolic starvation stress	Down	Induced	YTHDF2-mediated promotes melanoma tumorigenesis and anti-PD-1 resistance	[98]
Drug resistance in NSCLC	METTL3	ATG5/ATG7	Up	Positive	METTL3 positively regulated autophagy in gefitinib resistance	[19]
Drug resistance in HCC	METTL3	ATG5/ATG7/ ATG16L1	Down	Negative	METTL3-mediated FOXO3 mRNA stabilization and negative impact on ATG proteins in sorafenib resistance	[51]
Drug resistance in CGC	METTL3	SQSTM1	Up	Negative	Impaired autophagic degradation of lncRNA stabilizes ARHGAP5 mRNA via facilitating METTL3 in chemoresistance	[99]

*IVD* Intervertebral disc, *HCC* hepatocellular carcinoma, *NSCLC* non-small cell lung cancer, *CRC* colorectal cancer, *CGC* chemoresistant gastric cancer

Recently, Wang et al*.* conducted a study in leukocytes collected from chronic kidney disease (CKD) patients. In this study, leukocytes exhibited decreased m6A modification levels. The reason for this outcome was the increased demethylase activity of FTO [102]. The FTO-mediated downregulation of m6A could have influenced the autophagy process in leukocytes. The resulting impairment might have contributed to disrupting normal kidney functions because autophagy plays a critical role in the physiological functions of the kidney[103]. Therefore, it is necessary to identify molecules that could regulate m6A modification and autophagy to improve leukocyte functions in CKD.

m6A modification is the most significant internal epigenetic modification in eukaryotic mRNA and highly enriched in brain tissue [23, 104]. It is also reported that m6A could regulate the physiological functions of the mammalian nervous system, including synaptic plasticity, learning, and memory [105, 106]. A genome-wide association study (GWAS) revealed that m6A dysfunction is linked to developing neurological disorders[107]. Furthermore, several studies have confirmed the role of m6A modification in the development of these disorders [108, 109]. Altered m6A regulation could play an important role in the occurrence of Alzheimer’s disease and its associated dementia [110, 111]. Chen et al*.* revealed a critical role of m6A modification in developing Parkinson’s disease (PD) [112]. Qiu et al*.* reported that m6A-associated single nucleotide polymorphisms could increase the risk of PD incidence [113]. Literature review indicates that impaired autophagy could result in the aggregation of misfolded proteins, which is considered a hallmark of neurodegenerative diseases [114, 115]. Therefore, data have showed that both m6A modification and autophagy could play a substantial role in the onset of neurodegenerative diseases respectively. In the future, it is important to investigate the role of the m6A-autophagy axis in the incidence and progression of neurodegenerative diseases. It could help for better understandings in the development and treatment of neurological disorders.

The relationship between m6A and autophagy has been investigated in many human disorders, but findings are still limited to make comprehensive inferences. Further research is needed to decipher the exact role of m6A-autophagy interplay in the incidence of various pathological conditions. The resulting data could help understand molecular mechanisms exploited by m6A-autophagy interaction to induce human disorders. These findings could also offer the possibility of developing novel therapeutic strategies to overcome m6A-autophagy interaction mediated human disorders.

## Data Availability

Not applicable.

## Abbreviations

ALKBH5: Alpha-ketoglutarate-dependent dioxygenase alkB homolog 5
ALP: Autophagy-lysosomal pathway
AMPK: Adenosine 5-monophosphate-activated protein kinase
ATG: Autophagy-related
BMSCs: Bone marrow-derived mesenchymal stem cells
CAMKK2: Calcium/calmodulin-dependent protein kinase kinase 2
CGC: Chemo-resistant gastric cancer
CKD: Chronic kidney disease
CMA: Chaperone-mediated autophagy
CRC: Colorectal cancer
CVDs: Cardiovascular diseases
FIP200: Focal adhesion kinase family interacting protein of 200 kDa
FOXO3: Forkhead box class O3
FTO: Fat mass and obesity associated
HCC: Human hepatocellular carcinoma
HNRNPD: RNA binding proteins heterogeneous nuclear ribonucleoprotein D
IGF2BPs: Insulin-like growth factor 2 mRNA-binding proteins
IVD: Intervertebral disc
LAMP-2A: Lysosomal-associated membrane protein 2A
LCs: Leydig cells
lncRNAs: Long non-coding RNAs
m6A: N6-methyladenosine
METTL3: Methyltransferase-like 3
METTL14: Methyltransferase-like 14
METTL16: Methyltransferase-like 16
mTOR: Mammalian target of rapamycin
mTORC1: Rapamycin complex 1
NPCs: Nucleus pulposus cells
NSCLC: Non-small cell lung cancer
PI3K: Phosphatidylinositol 3-kinase
RBM15: RNA binding motif protein 15
TFEB: Transcription factor EB
ULK1/2: Unc-51 like kinase 1/2
WTAP: Wilms’ tumor 1-associating protein
YTHDC1-2: YTH domain-containing proteins 1-2
YTHDF1-3: YTH domain-containing family proteins 1-3
